# Multi-omics combined with MALDI mass spectroscopy imaging reveals the mechanisms of biosynthesis of characteristic compounds in *Tetrastigma hemsleyanum* Diels et Gilg

**DOI:** 10.3389/fpls.2023.1294804

**Published:** 2024-01-09

**Authors:** Yan Lin, Xuechun Jiang, Sheng Zhu, Junling Dun, Jinbao Pu, Weiqing Liang

**Affiliations:** ^1^ Department of Pharmacy, Tongde Hospital of Zhejiang Province, Hangzhou, China; ^2^ Key Laboratory of Research and Development of Chinese Medicine of Zhejiang Province, Zhejiang Academy of Traditional Chinese Medicine, Hangzhou, China; ^3^ Zhejiang Guangsheng Pharmaceutical Co., Ltd., Quzhou, China; ^4^ Analytical Applications Center, Shimadzu (China) Co., Ltd., Shanghai, China

**Keywords:** *Tetrastigma hemsleyanum*, MALDI mass spectrometry imaging, transcriptomics, proteomics, characteristic compound, biosynthesis mechanism

## Abstract

*Tetrastigma hemsleyanum* Diels et Gilg is recognized as a source of extracts with various desirable bioactivities. However, current knowledge regarding the mechanisms of biosynthesis of flavonoids, phenolic compounds, and other bioactive chemicals is limited. We conducted comprehensive tissue distribution studies and biosynthetic analyses of the 26 main bioactive compounds of this plant. The majority of flavonoids exhibited higher concentrations in the cortex (CT) compared to the vascular cylinder (VC). The expression levels of genes and proteins in CT and VC were quantified using mRNA sequencing and isobaric tags for relative and absolute quantification (iTRAQ). A total of 31,700 genes were identified, among which 4921 exhibited differential expression between CT and VC. A total of 13,996 proteins were identified in the proteomes of CT and VC, with 927 showing differential expression. Co-expression network analyses of DEGs and DEPs from multiple sites demonstrated substantial pathway variations linked to flavonoid biosynthesis. Through differential enrichment analysis, a total of 32 genes involved in the flavone biosynthesis pathway were identified, with iTRAQ specifically detecting C3’H, F3H and FLS. Pearson correlation analysis revealed a strong association between the expression levels of C3’H, F3H, and FLS and the concentrations of flavonoids. The validation of multiple genes encoding pivotal enzymes was conducted using real-time fluorescence quantitative PCR (RT-qPCR). The findings provide a foundation for future investigations into the molecular mechanisms and functional characterization of *T. hemsleyanum* candidate genes associated with characteristic compounds.

## 1 Introduction

The perennial herb *Tetrastigma hemsleyanum* Diels et Gilg, otherwise known as “san ye qing”, is renowned in Chinese herbal medicine for its rich content of flavonoids and phenols, which make it a valuable remedy for fever, pneumonia, and sore throat, and causing it to exhibit potential anti-tumor properties (Hu et al., 2021; Ji et al., 2021). Therefore, a comprehensive understanding of the biochemical foundations underlying the production of the principal active compounds of *T. hemsleyanum* would be valuable. To date, numerous compounds have been isolated from extracts of this plant, suggesting the potential diverse biological and pharmacological effects of the individual secondary metabolites (Hu et al., 2021; Ji et al., 2021). However, to date, no comprehensive investigation has been conducted to assess the biosynthetic sites and formation mechanisms of the major compounds in *T. hemsleyanum*.

Determining the spatial distribution of endogenous molecules is an important step in the investigation of mechanisms leading to the synthesis of plant metabolites. Specifically, uneven rates of metabolism across various cellular and subcellular compartments within plant tissues results in a heterogeneous distribution of plant metabolites (Lunn, 2007). Therefore, elucidating the spatial organization of metabolites in different tissues of *T. hemsleyanum* would shed light on the roles of these chemicals in plant growth as well as factors leading to chemical abundances. Plant imaging techniques, such as light and electron microscopy, are utilized for morphological characterization and the localization of labeled compounds within plant tissues (Gierlinger and Schwanninger, 2007; Kopittke et al., 2018). However, the utilization of these methods is subject to significant limitations. Therefore, an efficient method for *in situ* examination of the spatial distribution of endogenous molecules in *T. hemsleyanum* is needed. Matrix-assisted laser desorption/ionization time-of-flight mass spectrometry (MALDI-TOF-MSI) represents a cutting-edge molecular imaging technology (Li et al., 2023; Liu et al., 2023) that can be utilized for qualitative identification of numerous known or unknown compounds, and it enables the acquisition of spatial distribution information from tissue surfaces, making it suitable for sample distribution analysis in complex systems. It can be utilized to depict the distribution of compounds in tissues at the molecular level (Hou et al., 2022).

Transcriptome sequencing (RNA-Seq) is a highly efficient and extensively employed molecular biological analysis technique that accurately reflects the gene expression profile of an organism (Hamilton and Robin Buell, 2012). Proteomics, a fundamental discipline in biological science, investigates the abundances of proteins within tissues or whole organisms, leading to information regarding protein expression levels, the processes of translation and post-translational modifications, as well as interactions (Canovas et al., 2004). In particular, isobaric tags for relative and absolute quantification (iTRAQ) represents a prevalent quantitative technique (Wiese et al., 2007). The integration of transcriptome and proteome data is widely employed to provide a comprehensive understanding of an organism’s biological processes at both gene and protein levels, offering a holistic view of physiological regulatory mechanisms from diverse perspectives (Wu et al., 2021; Guerrero-Sánchez et al., 2022; Park et al., 2022).

**Figure 1:**
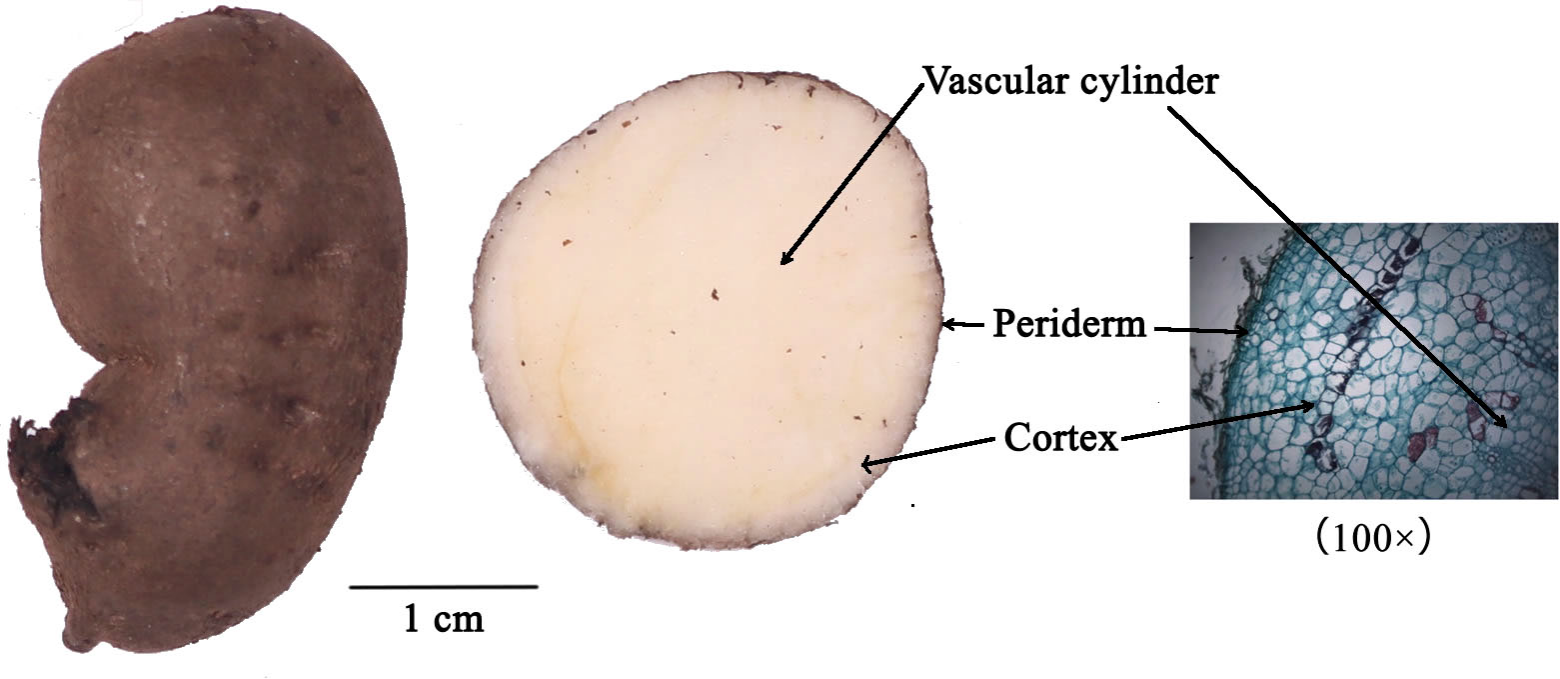
Sample information and transverse section of *T. hemsleyanum*.

In order to investigate differences in the abundances and mechanisms of formation of compounds in different parts of *T. hemsleyanum* root. We first employed MALDI mass spectrometry imaging to spatially map the distribution of endogenous molecules in *T. hemsleyanum*, followed by an analysis of their distribution patterns (**Figure 1**). The gene activity in different parts of cortex (CT) and vascular cylinder (VC) from *T. hemsleyanum* were analyzed using an integrated approach combining RNA-Seq and iTRAQ, enabling a comparison of mRNA and protein abundances. The comprehensive analysis of gene and protein expression data provided a holistic understanding of the synthesis of major compounds in *T. hemsleyanum*. The findings presented in this study expand and enhance our comprehension of the mechanisms underlying the formation of key compounds in *T. hemsleyanum*, while also identifying potential candidate genes that could contribute to enhancing *T. hemsleyanum* quality. These results offer a scientific foundation for breeding programs and regulatory efforts aimed at improving *T. hemsleyanum* crops.

## 2 Materials and methods

### 2.1 Plant material

*T. hemsleyanum* samples were planted in April 2017 and collected stable swelling roots in November 2019, from the seed site of Zhejiang Guangsheng Pharmaceutical Co Ltd, located in Qujiang District, Quzhou City, Zhejiang Province, China (28°53’6.216’’N, 118°47’45.744’’E). For each sample, a minimum of three plants were collected and processed as three independent biological replicates. The samples for mass spectrometry compositional analysis underwent RNA extraction and iTRAQ protein analysis. All samples were prepared, immediately frozen with liquid nitrogen, and stored in a -80°C freezer.

### 2.2 Mass spectrometry imaging analysis

#### 2.2.1 Selection of the MALDI matrix

**Figure 2:**
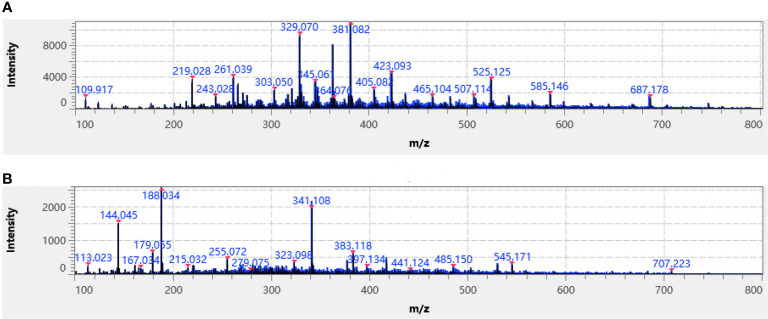
Average mass spectra of *T. hemsleyanum* of typical **(A)** CHCA-positive ion mode and **(B)** 1,5-DAN-negative ion mode. Identified metabolites are labeled with a red asterisk.

In the positive and negative ion detection mode, a comparative analysis was conducted using five commonly utilized substrates, 9-aminoacridine (9-AA), 2-[(2E)-3-(4-tert-butylphenyl)-2-methylprop-2-enylidene] malononitrile (DCTB), 2,5-dihydroxybenzoic acid (DHB), α-cyano-4-hydroxycinnamic acid (CHCA) and 1,5-diaminonaphthalene (1,5-DAN). An iMLayer AERO automatic matrix spray instrument was used to dispense the first four substrates as liquid matrixes at concentrations of 5 mg/mL, while the 1,5-DAN substrate was applied by manual airbrushing. Upon data acquisition, it was observed that using CHCA as the substrate in positive ion mode resulted in higher relative peak intensity of analyte (**Figure 2A**), while using 1,5-DAN as the substrate in negative ion detection mode showed higher relative peak intensities (**Figure 2B**). Therefore, for MALDI mass spectrometry imaging of *T. hemsleyanum*, CHCA was chosen as the matrix for the positive ion detection mode and 1,5-DAN was chosen as the matrix for the negative ion detection mode.

#### 2.2.2 Sample preparation

The samples were immersed in an aqueous gelatin solution with a concentration of 0.1 g/mL, followed by rapid freezing in liquid nitrogen. Frozen slices (40μm) were obtained from the cryosections (Lycra CM1950) of the frozen embedded samples. The frozen slices were maintained at a temperature of -20°C during the experiment. Subsequently, the frozen slices were affixed onto indium tin oxide slides using conductive double-sided adhesive and subsequently desiccated under vacuum conditions. The CHCA substrate was vapor-deposited onto the sample surface using a Shimadzu iMlayer substrate sublimator, resulting in a thickness of 0.7 μm. Additionally, the 1,5-DAN substrate was applied by manual spraying using a spray gun (PS-270, GSI Creos) equipped with a 0.22 mm nozzle. Finally, the substrate-coated slides were sent for analysis on an imaging mass spectrometry microscope.

#### 2.2.3 Mass spectrum imaging and MS/MS analysis

The MALDI-TOF-MS imaging data were acquired using an iMScope TRIO imaging mass spectrometer (Shimadzu) equipped with a built-in light microscope and a 355 nm Nd: YAG laser source. Ions within the m/z range of 100 to 800 were measured in both positive and negative ionization modes. The laser diameter was set to 1 (an arbitrary unit of iMScope that is approximately equal to 10 μm). Other relevant parameters for MALDI-TOF-MS imaging were as follows: the detector voltage was 2.50 kV, the sample voltage was 3.50 kV, the radiation was 700 times, the scanning frequency was 2000 Hz, the laser energy was 68, the pixel pitch was 20 × 20 μm^2^, and the laser intensity was 20 arbitrary units. Further ion identification was performed using MS/MS on a tissue section of *T. hemsleyanum* with argon used as the collision gas and corresponding precursor ions subjected to collision-induced dissociation (CID) to obtain different product ions. In the positive ionization mode, normalized collision energy was set between 16% to 25%. The molecular images were acquired and analyzed using Imaging MS Solution version 1.30 software (Shimadzu).

#### 2.2.4 Region of interest analysis

Region of interest (ROI) analyses were conducted on various sections of *T. hemsleyanum* using Imaging MS Solution version 1.30 software (Shimadzu). ROI were selected by using data and corresponding optical images from mass spectrometry imaging analyses so that the analyses included multiple parts of the *T. hemsleyanum* with m/z values in the 100 to 800 range. The average mass spectra of analyzed compounds were also measured to determine the average signal intensity from m/z 100 to 800. The Mann-Whitney U-test was employed to evaluate significant differences in peak intensities between sites.

### 2.3 RNA extraction, sequencing, and *de novo* assembly

Total RNA was extracted from *T. hemsleyanum* root tissue using TRIzol^®^ Reagent (Plant RNA Purification Reagent for Plant Tissue; Invitrogen, Carlsbard, CA, USA) according the manufacturer’s instructions, and genomic DNA was removed using DNase I (TaKara). The integrity and purity of the total RNA was determined using a 2100 Bioanalyser (Agilent Technologies, Inc., Santa Clara CA, USA) and quantified using a NanoDrop ND-2000 spectrophotometer (Thermo Scientific, Wilmington, DE, USA). Only high-quality RNA samples (OD_260_/OD_280 = _1.8 - 2.2, OD_260_/OD_230_ ≥ 2.0, RIN ≥ 8.0, 28S:18S ≥ 1.0, and >2μg) were used to construct the sequencing library.

The TruSeq™ RNA Sample Preparation Kit (Illumina, San Diego, CA, USA) was utilized for library preparation. The isolated mRNA was fragmented into approximately 300 bp fragments using a fragmentation buffer, and these short fragments were used as templates to synthesize double-stranded cDNA with the SuperScript Double-Stranded cDNA Synthesis Kit (Invitrogen, Carlsbad, CA, USA) and random hexamer primers (Illumina, San Diego, CA, USA). End repair was performed according to the Illumina library construction protocol. After quantification with TBS380, two RNAseq libraries were single-line sequenced on an Illumina Hiseq xten/NovaSeq 6000 sequence reader (Illumina, San Diego, CA, USA) to obtain 2 × 150 bp paired-end reads at Shanghai Majorbio Biomedical Technology Co., LTD. (Shanghai, China). Raw paired-end reads were trimmed and quality controlled using SeqPrep and Sickle with default parameters. The purified transcriptome data was *de novo* assembled using Trinity software.

### 2.4 Protein extraction, iTRAQ labeling and quantification

The protein extraction, iTRAQ labeling, and quantification of all samples were conducted by Shanghai Majorbio Biomedical Technology Co., LTD. (Shanghai, China). Lysates of root tissue were obtained by grinding in liquid nitrogen followed by suspension in borax/polyvinylpolypyrrolidone/phenol (BPP) buffer containing 100 mM EDTA, 50 mM borax, 50 mM ascorbic acid, 30% sucrose, 100 mM TrisBase, 1% TritonX-100, 1 g/L polyvinylpolypyrrolidone, and 5 mM Dithiothreitol at pH 8.0. Subsequently, an equal volume of Tris-saturated phenol was added, followed by stirring for 10 min at 4°C. The mixture was then centrifuged at 12,000 *g* for 20 min at 4°C, and the phenol phase was combined with an equal volume of BPP buffer and stirred for 10 min at 4°C.

After centrifugation at 12,000 *g* for 20 min at 4°C, the phenol phase was supplemented with a 5-fold volume of pre-cooled methanolic ammonium acetate solution to precipitate proteins at -20°C for twelve hours. The dry precipitate was subjected to two washes with lysis buffer containing 1% SDS and 8 mol/L urea, followed by sonication in an ice bath for three minutes. The lysate was then centrifuged at 12,000 *g* for 20 min at 4°C, and the resulting supernatant was collected. Protein concentrations were determined using the BCA Protein Assay Kit (Thermo Fisher Scientific, Waltham, MA, USA).

Peptide sequencing was performed essentially as described (Zeng et al., 2019). Briefly, proteins were digested with trypsin (Promega, Madison, WI, USA) and subsequently labeled with 8-plex iTRAQ reagent (Sciex, Foster, CA, USA). To enhance protein isolation and increase proteome depth, an ACQUITY Ultra Performance Liquid Chromatography (Waters, Milford, MA, USA) equipped with a BEH C_18_ column (2mm × 150mm, 1.7µm; Waters, Milford, MA, USA) was employed. Finally, LC-MS/MS analysis was conducted using an EASY-nLC system coupled to a high-resolution mass spectrometer (Thermo Fisher Scientific, Waltham, MA, USA) featuring a nano-electrospray ion source.

### 2.5 Correlation analysis of the transcriptome and proteome

The expression abundance of each individual gene was quantified using the RNA-Seq by Expectation-Maximization (RSEM) algorithm, which provided values in fragments per kilobase of transcript per million mapped reads (FPKM). Raw counts were subjected to analysis using DESeq2 software, employing a negative binomial distribution with |log_2_FC| ≥ 1 and FDR value < 0.01. Differential expression was determined based on fold-change criteria greater than 1.2 for up-regulated proteins or less than or equal to 0.83 for down-regulated proteins, with a significance level of *p* < 0.01.

### 2.6 Bioinformatics analysis

Differentially expressed genes (DEGs) and differentially expressed proteins (DEPs) were functionally classified using Blast2GO (https://www.blast2go.com/b2ghome) based on gene ontology (GO) terms (https://www.geneontology.org), and enriched metabolic pathways were annotated with *p* < 0.01 through the KEGG database (https://www.genome.jp/kegg/).

### 2.7 Reverse transcription-quantitative PCR analysis

The gene-specific primers, as listed in **Table 1**, were designed and synthesized by Shanghai Sangon Bio-technology Co., Ltd. (Shanghai, China). Total RNA was extracted from the samples using the OminiPlant RNA Kit (DNase I) (Cowin Bio, CW2598S), following the manufacturer’s standard protocol. The cDNA was obtained using the FastKing RT Kit (With gDNase) (TIANGEN, KR116-02) from TIANGEN Biothech (Beijing, China). PCR amplification was performed using SuperReal PreMix Plus (SYBR Green) (TIANGEN, FP205-02) with a reaction volume of 20 μL. Each reaction contained 2 μL of cDNA, 10 μL of 2× SuperReal PreMix Plus, 0.6 μL of each forward and reverse primer (10 μmol/L), 2 μL of 50× ROX Reference Dye^△^, and 4.8 μL of ddH_2_O. The RT-qPCR cycling conditions consisted of an initial denaturation at 95°C for 15 min followed by 40 cycles of denaturation at 95°C for 10 s and annealing/extension at 60°C for 10 s. A melting curve analysis was then performed. Gene expression levels were normalized to GAPDH as an internal control gene and calculated as fold changes using the ΔΔCt method with three biological replicates (Livak and Schmittgen, 2001).

**Table 1:** Primers sequences used for RT-qPCR for detecting gene expression.

Gene	Primer F (5’-3’)	Primer R (5’-3’)
CYP73A	AATCCAACCTCCACCTACT	CGCATTCACCACTACCTT
CHS	ATAATCAGCACCAGGCATT	TACGCCAAGAGATCATCAC
C3’H	GGCAGTCACTTCATCTTCT	TATGGACCTCACTACATCAAG
F3H	ACTCACCGCTATCAACCA	GCCTCCACAATCTTCCTAC
FLS	GATAGTTAATGGCGGAAGGA	GCAGGAGGAGAAGGAAGT
CYP75A	CACTTCATCCAGCATAATCG	GTAGCCGTTCACTTCACA

### 2.8 Statistical analysis

Differences were analyzed using ANOVA or Pearson correlation analyses. Differences for which *p* < 0.05 were considered statistically significant.

## 3 Results

### 3.1 Mass spectrometry imaging of root sections from *T. hemsleyanum*

**Figure 3:**
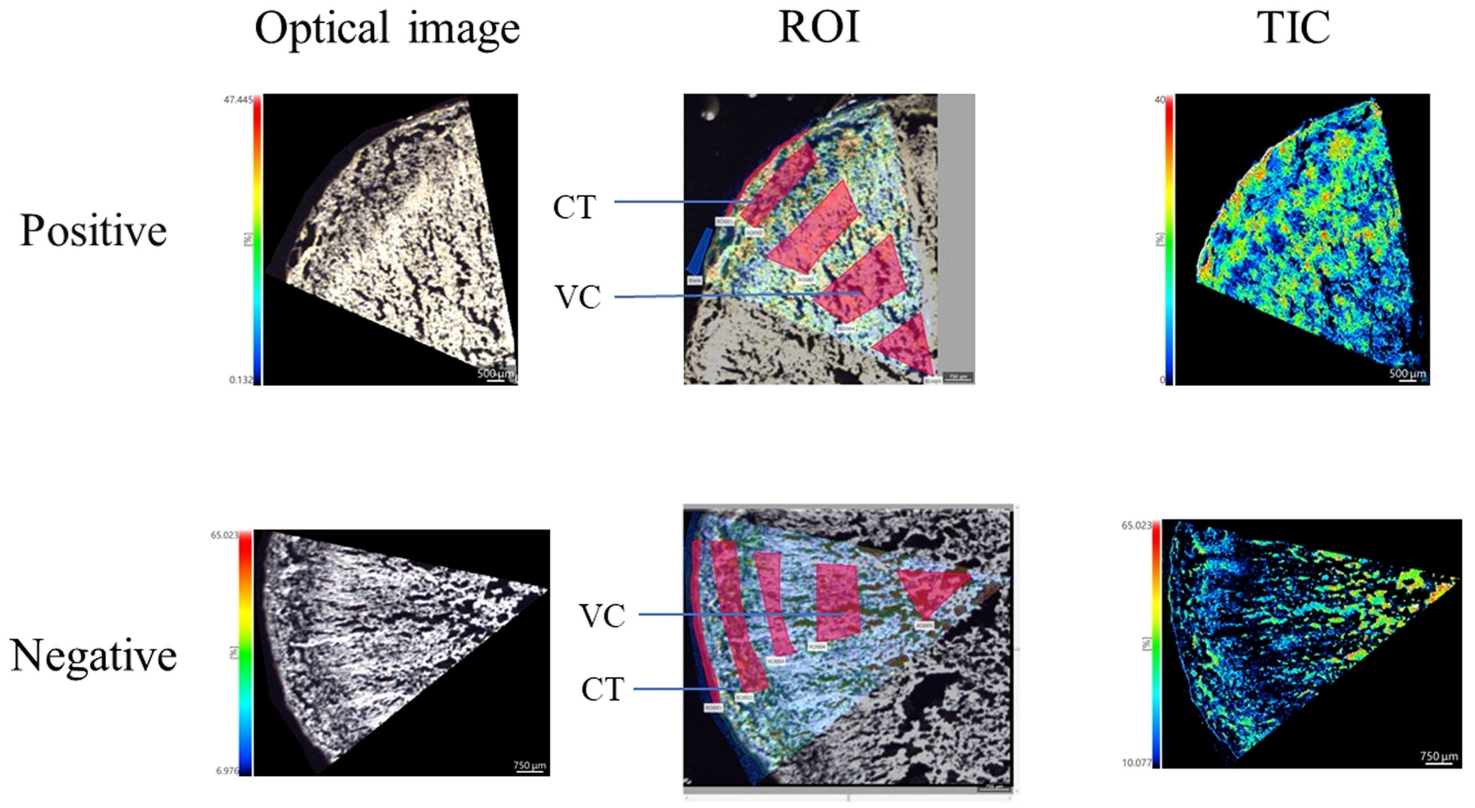
MALDI mass spectrometry imaging results and the light microscope of *T. hemsleyanum* on the cross section.

Mass spectrometry was performed in was performed in positive ion mode using CHCA as the matrix and in negative ion mode using 1,5-DAN as the matrix. The cross section of *T. hemsleyanum* was subjected to analysis over an m/z range of 100 to 800. Mass spectra of the samples that illustrate their average characteristics are shown in **Figure 2**. Optical images, along with ROI settings and total ion chromatograms (TIC) of the corresponding slices, are shown in **Figure 3**.

The results of the quantitative analysis, obtained through application of IMAGEREVEAL MS software and in conjunction with previous high-resolution mass spectrometry studies as well as relevant literature reports, are presented in **Supplementary Table S1**. The main flavonoids identified in *T. hemsleyanum* were quercetin, catechin, and hybridin (structural information see **Supplementary Figure S1**). *In situ* visualization analysis using iMScopeQT revealed a higher abundance of most flavonoids in CT compared to VC (**Supplementary Figure S2**). The concentration of flavonoids in the CT was found to be higher in the vicinity of the VC, while a gradual decrease in flavonoid content was observed from the outer to inner regions of the VC.

The spatial distribution trends of several compounds exhibited variations, such as the higher abundance of kaempferol-3-O-*β*-D-(2”-O-*β*-D-apiofuranosyl) glucopyra-noside-7-O-*α*-L-rhamnopyranoside in VC compared to CT. Salicylic acid, protocatechuate, quinic acid, and coumaroylquinic acid were identified as the predominant phenolic acids in the CT. The high levels of flavonoids present in the epidermis of *T. hemsleyanum* roots may be attributed to the plant’s inherent self-protective mechanism against a diverse range of biological and environmental stressors.

### 3.2 RNA sequencing data, *de novo* assembly, and proteome characterization

The transcriptomes and proteomes of CT and VC from *T. hemsleyanum* were analyzed in order to investigate the mechanisms of growth regulation of this plant species. The RNA-Seq findings are displayed in **Table 2**.** **A total of six samples, which included samples isolated in triplicate from two parts of the plant (CT and VC), underwent transcriptome sequencing. In this process, 305,171,170 clean reads comprising 45.06 Gb of clean bases were obtained after filtering the initial set of 308,486,874 raw reads. Based on the obtained high-quality bases, the average Q20 and Q30 values of the six samples were 97.58% and 93.71%, respectively.

**Table 2:** Quality assessment of the RNA-Seq data.

Sample	Raw reads	Clean reads	Error rate(%)	Q20(%)	Q30(%)	GC content(%)
CT_1	42058422	41514848	0.03	97.52	93.68	46.78
CT_2	49052052	48521486	0.03	97.50	93.53	46.42
CT_3	52212900	51645814	0.03	97.57	93.76	46.91
VC_1	51842770	51379276	0.03	97.75	93.99	46.12
VC_2	56314734	55682798	0.03	97.57	93.68	46.47
VC_3	57005996	56426948	0.03	97.58	93.59	46.31

**Figure 4:**
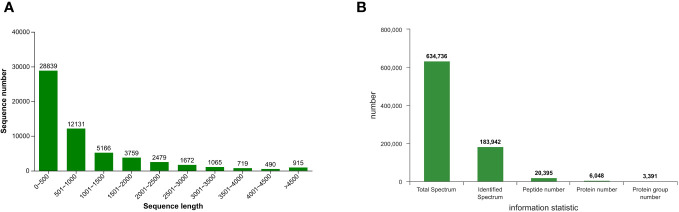
Sequence length distribution of the assembled **(A)** transcripts and **(B)** protein spectrum information of *T. hemsleyanum*.

A total of 57,235 single genes and 89,992 transcripts were obtained through *de novo* assembly of clean data, with an average length of 948.20 bp and an N50 value of 1733 bp. The distribution of lengths of assembled transcripts is depicted in **Figure 4A**, wherein the most abundant transcripts (28,839) exhibit a shorter length ranging from 0 to 500 bp, constituting approximately 50% of the entire assembly. Conversely, only 490 transcripts possess lengths between 4001 and 4500 bp. Proteomic analysis identified a total of 20,395 peptides, 6048 proteins, and 3391 protein groups across the samples (FDR < 0.01) (**Figure 4B**), providing robust evidence for the reliability of both RNA-seq and iTRAQ techniques and the availability of corresponding data.

### 3.3 Principal component analysis for comparative analysis of transcriptome and proteome data

**Figure 5:**
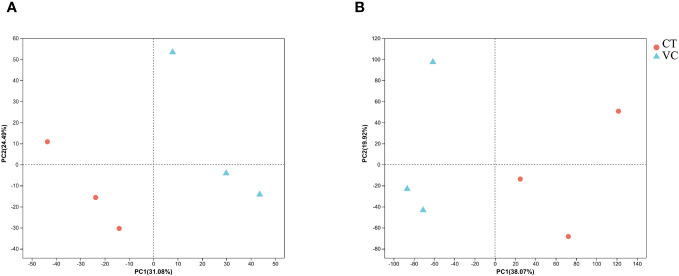
The PCA analysis for **(A)** genes and **(B)** proteins expression of *T. hemsleyanum*.

Principle component analyses (PCA) were performed on both transcriptome and proteome profile data. The samples in both plots exhibited distinct clustering patterns, with clear differentiation observed among the clusters representing the three biological replicates of endothelial vascular bundles (**Figure 5**). This observation implies substantial variations in gene expression at the levels of mRNA and protein across different regions (CT and VC) of *T. hemsleyanum*, which explains the divergent metabolic profiles observed among these regions.

### 3.4 Correlation analysis of DEGs and DEPs

#### 3.4.1 Venn analysis

**Figure 6:**
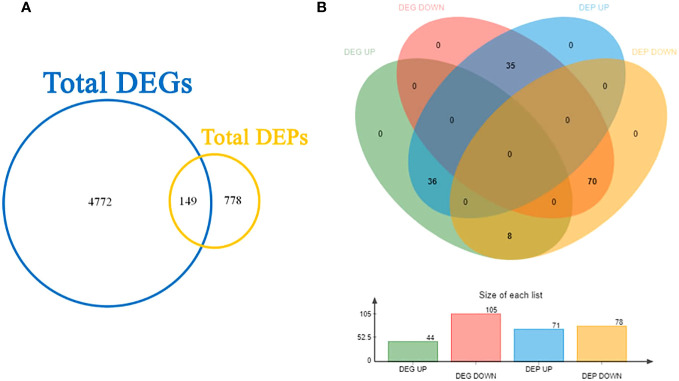
Joint Venn analysis for **(A)** total DEGs/DEPs and **(B)** shared factors in *T. hemsleyanum*.

Upon conducting transcriptome and proteome differential expression analysis, a total of 4921 DEGs and 927 DEPs were identified between the CT and VC (**Figure 6A**). Among these factors, at the transcript level, the levels of 44 genes were found to be higher in CT relative to VC, while the levels of 105 genes were found to be lower in the CT relative to VC. At the protein level, a total of 71 proteins were found to be up-regulated in CT, while 78 proteins exhibited down-regulation (**Figure 6B**).

Furthermore, when a Venn analysis was performed to compare relationships between gene expression at the mRNA level to that at the protein level, a total of 149 genes were found to exhibit consistent trends when comparing mRNA levels to protein levels. Conversely, for 106 genes, the trends were different for the mRNA and protein levels. Specifically, for 36 genes, the mRNA levels were higher than the protein levels, whereas for 70 genes, the mRNA levels were lower than the protein levels.

#### 3.4.2 GO Functional annotation

**Figure 7:**
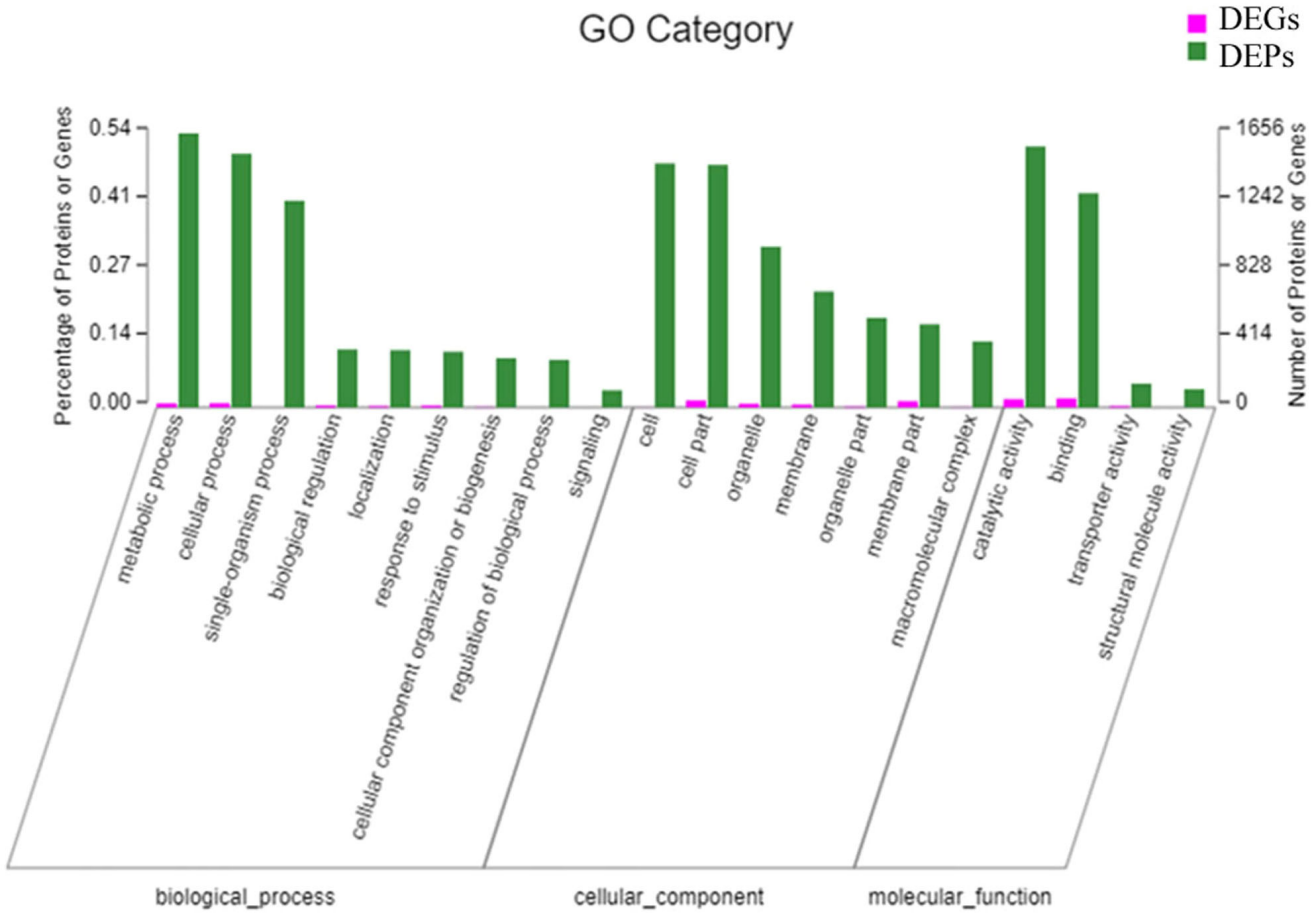
The top 20 GO annotated entries containing the largest DEGs/DEPs in *T. hemsleyanum*.

To investigate molecular mechanisms of secondary metabolism in *T. hemsleyanum*, we utilized GO to perform functional annotation analysis on DEGs and DEPs across three GO branches. The top 20 GO annotation entries are presented in **Figure 7**. In the category of biological processes, the DEGs and DEPs were primarily involved in metabolic, cellular, and single-organism processes. In terms of cellular and molecular functions, DEGs/DEPs were primarily involved in various aspects of cell biology, including cellular compounds, subcellular structures and organelles. In terms of cellular compounds, DEGs/DEPs were predominantly associated with catalytic activity and binding functions. The biosynthesis of the secondary metabolite pathway exhibited the highest enrichment for a substantial number of DEPs, with a total count of 1656 DEPs. The high representation of DEPs in biosynthesis of secondary metabolic pathways suggests that these pathways are particularly important for defining differences between the various functional tissues within the root. This conclusion is supported by the well-known involvement of metabolic processes in myriad factors, including growth and development (Wang et al., 2022) and tissue architecture maintenance (Prasongsansuk et al., 2020).

#### 3.4.3 KEGG pathway enrichment

**Figure 8:**
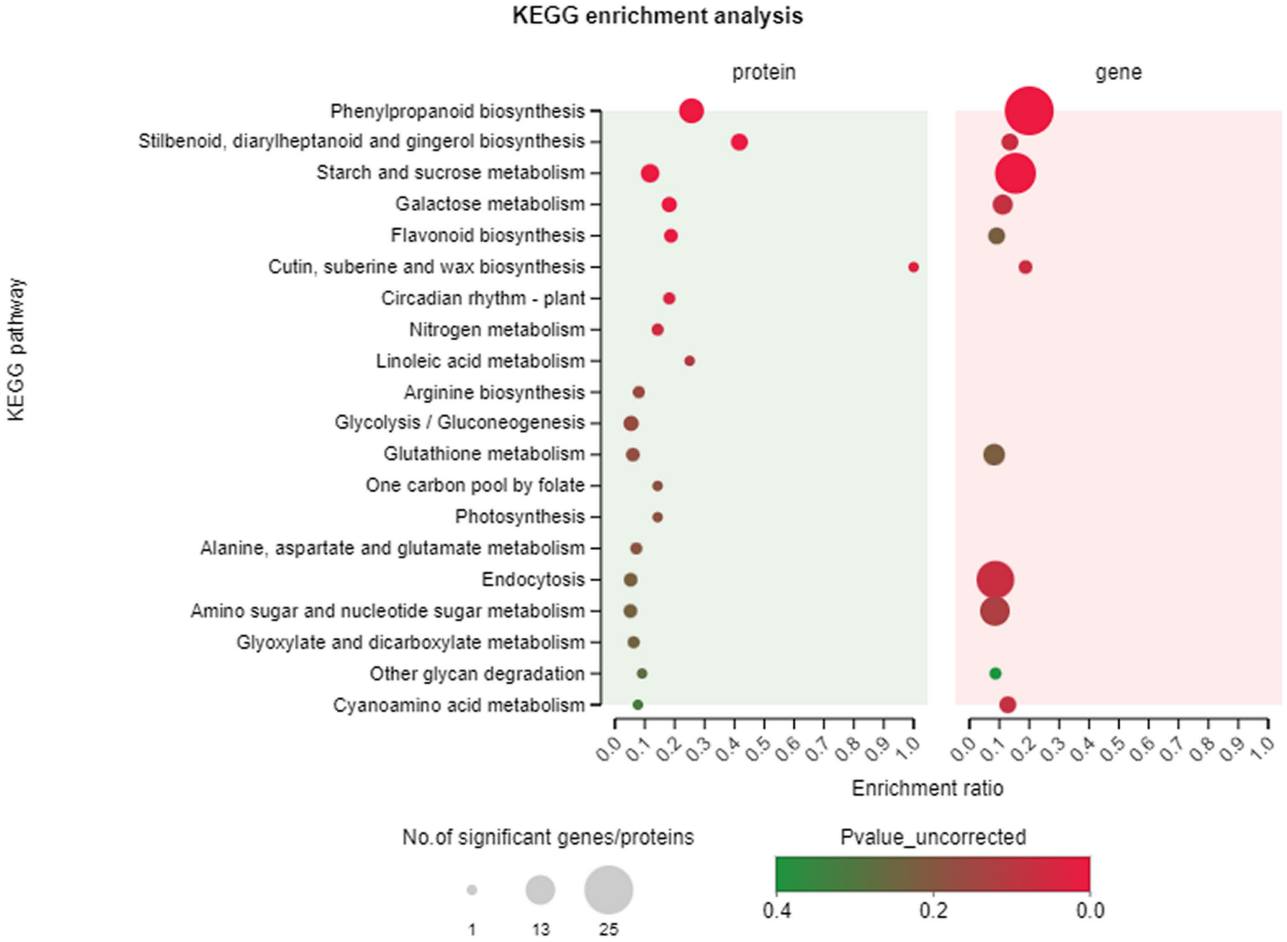
KEGG pathway enrichment analysis. The top 20 pathways of DEGs/DEPs in *T. hemsleyanum* according to the enrichment degree.

The top 20 KEGG pathways with the highest number of DEGs and DEPs were identified through a KEGG pathway enrichment analysis of the two data sets. As depicted in **Figure 8**, the pathways of phenolics biosynthesis, starch and sucrose metabolism, and endocytosis, and this figure also demonstrates that the gene and protein levels exhibited significant enrichment and commonality. The findings of this study suggest that the biosynthesis of flavonoids plays pivotal roles in specific anatomical regions of *T. hemsleyanum*.

### 3.5 Comprehensive analysis of flavonoid biosynthetic pathways

**Figure 9:**
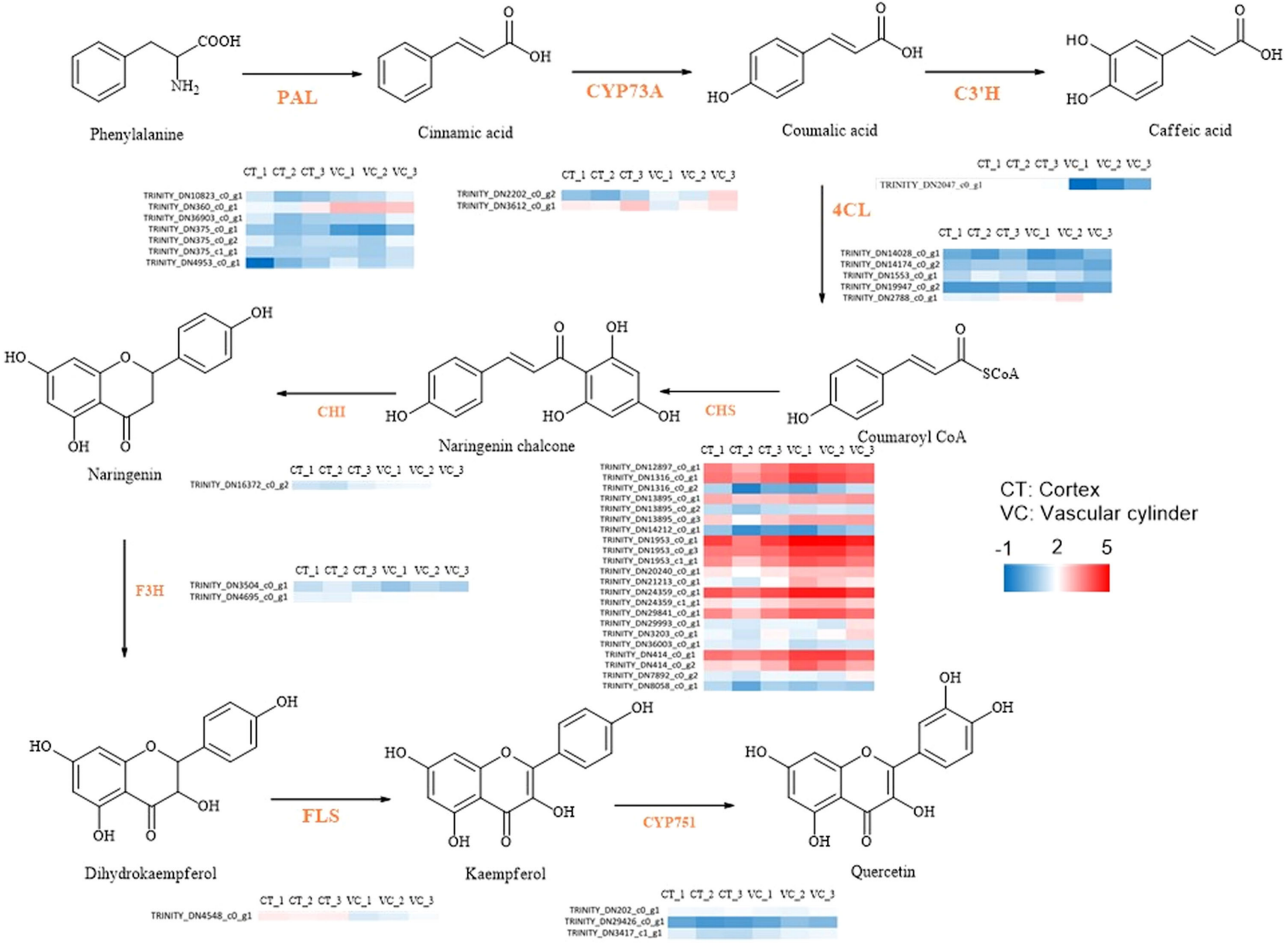
The DEGs associated with flavonoid biosynthesis in CT and VC of *T. hemsleyanum*. The red represented up-regulated DEGs. The blue represented down-regulated DEGs.

Through a comprehensive analysis, it was discovered that the DEGs and DEPs in the roots of *T. hemsleyanum* were involved in seven KEGG reference pathways, including phenolics biosynthesis, flavonoid biosynthesis and stilbenoid biosynthesis. This analysis thus revealed the patterns of secondary metabolite biosynthesis in the roots of *T. hemsleyanum*. The pathway of flavonoid metabolism, as depicted in **Figure 9**, encompasses nine pivotal genes implicated in the biosynthesis of characteristic compounds of *T. hemsleyanum* and major flavonoid metabolites. The heat map of synthetase expression revealed that genes involved in downstream steps in phenolics biosynthesis, including genes encoding chalcone synthase (CHS), p-coumarate 3-hydroxylase (C3’H), flavanone 3-hydroxylase (F3H) and flavonol synthase (FLS), were down-regulated in CT relative to VC. As a result, flavonoids accumulated at higher levels in the CT. The expression of the gene encoding phenylalanine ammonia-lyase (PAL) was found to be up-regulated at the mRNA and protein levels in the epidermis, leading to enhanced catalysis of trans-cinnamate into phenylalanine.

**Figure 10:**
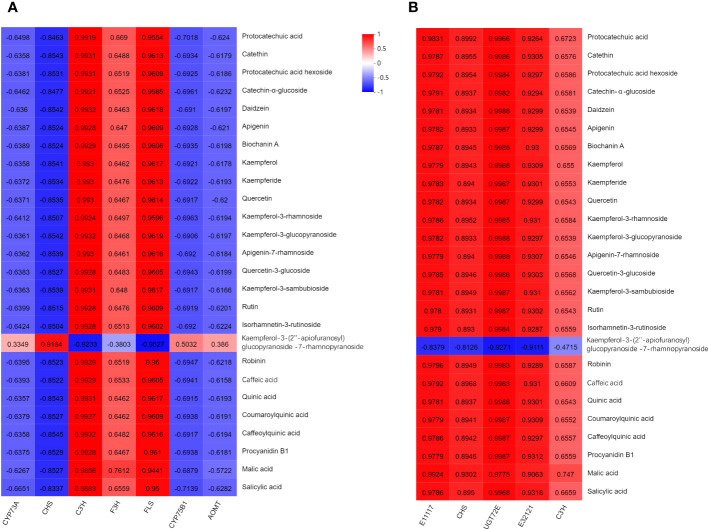
Correlation between major compounds, and **(A)** gene expression/**(B)** protein expression of related enzymes of *T. hemsleyanum*. The red represented positive correlation. The blue represented negative correlation.

To gain a comprehensive understanding of the regulatory network governing *T. hemsleyanum*, we conducted Pearson correlation analysis for each of the 7 genes, 6 proteins, and 26 metabolites involved in the flavonoid biosynthesis pathway (**Figure 10**). The results revealed 182 significant correlations between genes and metabolites, with a Pearson correlation coefficient < 0.8, *p* < 0.05. The regulatory networks significantly associated with all metabolites were constructed using a total of 7 genes and 5 proteins. The gene expression correlation analysis revealed significant positive associations of C3’H, F3H, and FLS with the principal compounds. The expression levels of the genes encoding trans-cinnamate 4-monooxygenase (CYP73A), CHS, flavonoid 3’-monooxygenase (CYP75B1), and flavonoid O-methyltransferase (AOMT) exhibited negative correlations with the major compounds.

In terms of protein expression analysis, there were significant positive associations observed between the levels of peroxidase (E1.11.1.7), CHS, coniferyl-alcohol glucosyl transferase (UGT72E), beta-glucosidase (E3.2.1.21), C3’H and the levels of the main compounds. Among them, only kaempferol-3-O-*β*-D-(2”-O-*β*-D-apiofuranosyl) glucopyranoside-7-O-*α*-L-rhamnopyranoside exhibited an inverse correlation.

### 3.6 Validation of the expression of genes involved in flavonoid accumulation

**Figure 11:**
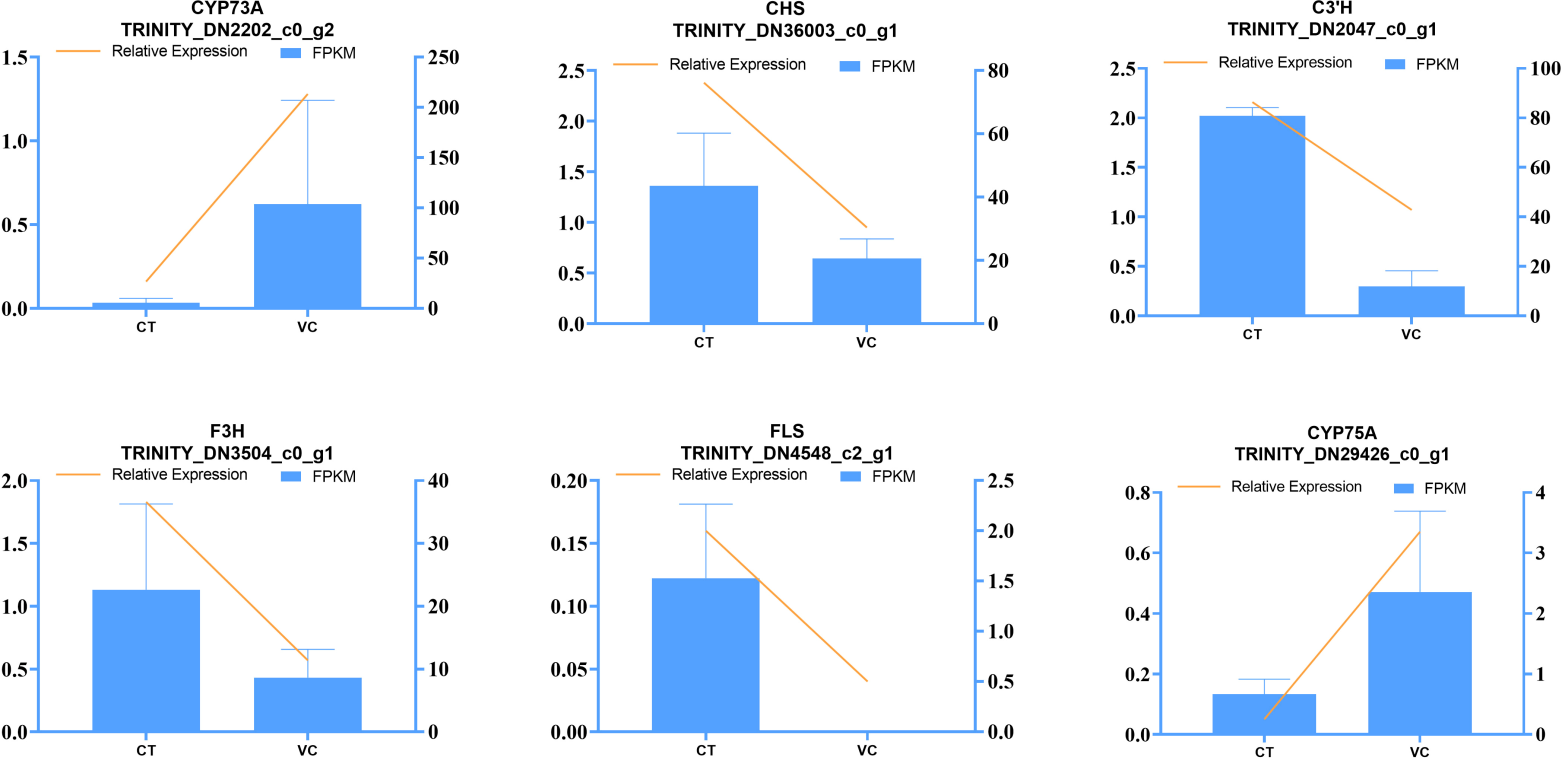
Verification of RNA-Seq sequencing data by the RT-qPCR assay. The left axis shows the relative expression, the right axis shows the fragments per kilobase million (FPKM) value, and the error line shows the SD value of the relative gene expression.

To validate the expression profile of DEGs identified by RNA-seq, we conducted RT-qPCR analyses on six DEGs in the CT and VC of *T. hemsleyanum* roots (**Figure 11**). The key genes identified in the transcriptome analyses to be involved in flavonoid synthesis were CHS (TRINI-TY_DN36003_c0_g1), C3’H (TRINITY_DN2047_c0_g1), F3H (TRINI-TY_DN3504_c0_g1), and FLS (TRINITY_DN4548_c2_g1). Therefore, the expression levels of these genes were quantified using RT-qPCR. The expression levels of these four target genes were found to be significantly higher in the CT as compared to the VC, consistent with the results of transcriptomic analyses. Moreover, notable decreases of the expression of CYP73A (TRINITY_DN2202_c0_g2) and CYP75A (TRINI-TY_DN29426_c0_g1) were observed in CT as compared to VC. The consistency of these results with those of the transcriptome analyses indicates that both the sampling methodology and RNA-seq technology employed in this study were appropriate for investigating the transcriptomic patterns of *T. hemsleyanum*.

## 4 Discussion

In the present study, we employed mass spectrometry imaging to investigate the distribution of characteristic compounds in *T. hemsleyanum* root, and we discovered that these compounds are present in higher concentrations within the CT as compared to VC. In contrast to the flavonoids found in *T. hemsleyanum* root, flavonoids and flavonoid glycosides are primarily synthesized within the CT. In addition, the CT were found to contain a multitude of compounds with known anti-inflammatory and anti-tumor effects, including catethin, daidzein, kaempferol, quercetin, robinin, caffeic acid, and quinic acid. Notably, quercetin and quinic acid were found to be the predominant compounds.

Transcriptomic and proteomic analyses of the CT and VC of *T. hemsleyanum* demonstrated a concordance between protein levels and the levels of their corresponding transcripts specifically. We detected transcripts of 83.31% of the detected proteins. We believe that many of the 484 transcripts that were not detected in this context are mRNA molecules of relatively low stability and short half-life. Interestingly, a total of 7568 transcripts lacking protein counterparts were identified in the transcriptome, suggesting their potential roles as transcriptional regulators. Consequently, a modest level of concordance (24.87%) was observed between the transcriptome and proteome, highlighting potential discrepancies in the transmission of genetic information from DNA to the ultimate phenotype (Sarethy and Saharan, 2021; Zheng et al., 2022). Moreover, this disconnect may also reflect the idea that protein abundance is subject to translational and post-translational processes that contribute to the dynamic nature of proteomes (Mann and Jensen, 2003).

When comparing the transcriptomes of the CT to the VC, multiple DEGs were observed, with more genes being upregulated in the CT relative to the VC. However, the opposite trend was observed when analysing the proteomic data. Many of these genes and proteins participate in metabolic pathways that encompass the biosynthesis of secondary metabolites as well as flavonoid biosynthesis. This enrichment of metabolic and flavonoid biosynthetic processes was identified through GO classification by association analysis of the DEGs and DEPs. It is plausible to hypothesize that a multitude of physiological factors, in conjunction with genes and proteins implicated in the biosynthesis of flavonoids, govern the observed metabolic disparities and would impact DEGs and DEPs across various parts of *T. hemsleyanum*.

Flavonoids, crucial secondary metabolites of *T. hemsleyanum*, are predominantly synthesized via the phenylpropane biosynthetic pathway (Yang et al., 2022; Zhu et al., 2023). In the present study, our comparative analysis of transcripts and metabolites across different anatomical regions of *T. hemsleyanum* tuber root led to the identification of 55 DEGs that were assigned to the flavonoid biosynthesis pathway. The majority of genes associated with flavonoid accumulation in the phenolics biosynthesis pathway exhibited up-regulation in CT (**Figure 9**), indicating a promotion of flavonoid biosynthesis within this segment. Numerous studies have demonstrated the involvement of transcription factors, including C3’H, F3H, FLS, CYP73A, CHS, CYP75B1 and AOMT, in the regulation of genes related to flavonoid biosynthesis. Accordingly, the activation and spatiotemporal expression of structural genes in flavonoid biosynthesis are determined by transcription factors and their interactions. Although enzymes with multiple subunits, each of which is encoded by a different gene, the observed disparity between DEGs and DEPs may be attributed to variations in mRNA synthesis rates, post-transcriptional regulation, as well as translational and post-translational mechanisms.

Based on the validation of gene expression through RT-qPCR, we postulated that DEGs in the flavonoid pathway are likely responsible for variations in flavonoid composition across different parts of *T. hemsleyanum* tubule root. This conclusion was supported by the results of mass spectrometry imaging analysis, which suggested that the differential enrichment of flavonoids could be attributed to the higher flavonoid content in CT and the up-regulated expression of C3’H, F3H, CHS and FLS in CT. A KEGG enrichment analysis revealed that flavonoids in the CT and VC are associated with multiple pathways, including flavonoid biosynthesis pathways, but also phenolics biosynthesis pathways, secondary metabolite biosynthesis pathways, and other pathways. DEGs in these pathways may encode pivotal enzymes or their regulators, thereby contributing to variations in flavonoid metabolite concentrations between the CT and VC.

Our results demonstrate the differential production and accumulation of multiple types of flavonoids, including anthocyanins, catechins, and quercetins, in different parts of the plant. In conclusion, this integrated transcriptomic and proteomic analysis of distinct sections of the *T. hemsleyanum* tuber root suggests the presence of important dynamic physiological factors governing flavonoid biosynthesis. The findings presented here provide a foundation for the development of superior *T. hemsleyanum* cultivars with elevated levels of bioactive compounds that could be produced by genetically altering the expression of relevant genes.

## 5 Conclusions

This study demonstrates the successful visualization of the spatial distribution of major compounds in the *T. hemsleyanum* tuber root using MALDI-MSI. Optimization of the MALDI matrix enabled imaging of nineteen flavonoids and phenols from *T. hemsleyanum*. Thereby facilitating a comprehensive understanding of the physiological functions and molecular mechanisms underlying *T. hemsleyanum* secondary metabolism while providing valuable insights that lay the basis for follow-up studies. The correlation analysis of the transcriptome and proteome revealed a total of 149 factors that were represented in both the DEGs and DEPs of a given sample. Among these, 44 genes and 71 proteins were found to be significantly up-regulated in the CT, while 105 genes and 78 proteins were down-regulated. The results indicate that differential regulation of the pathways leading to the biosynthesis of phenols and flavonoids underlies the variation in metabolite profiles across different regions of the *T. hemsleyanum* tuber root, shedding light on the physiological significance of endogenous molecules in *T. hemsleyanum* and providing a valuable molecular foundation for investigating *T. hemsleyanum* metabolite biosynthesis and accumulation.

## Data availability statement

The original contributions presented in the study are publicly available. This data can be found here: https://www.ncbi.nlm.nih.gov/bioproject/PRJNA1015019.

## Author contributions

YL: Methodology, Project administration, Writing – original draft. XJ: Methodology, Software, Writing – review & editing. SZ: Resources, Writing – review & editing. JD: Data curation, Methodology, Visualization, Writing – review & editing. JP: Project administration, Supervision, Writing – review & editing. WL: Data curation, Formal Analysis, Writing – original draft.
