# Supplementary material for: Multi-omics combined with MALDI mass spectroscopy imaging reveals the mechanisms of biosynthesis of characteristic compounds in Tetrastigma hemsleyanum Diels et Gilg
Source: Front Plant Sci. 2024 Jan 9;14:1294804. doi: 10.3389/fpls.2023.1294804 (PMC10803607; doi:10.3389/fpls.2023.1294804)
Supplement: Supplementary file 1 [file DataSheet_1.docx]

Supplementary Material

**Supplementary Figure S1.** The structures of 26 compounds in *T. hemsleyanum*.


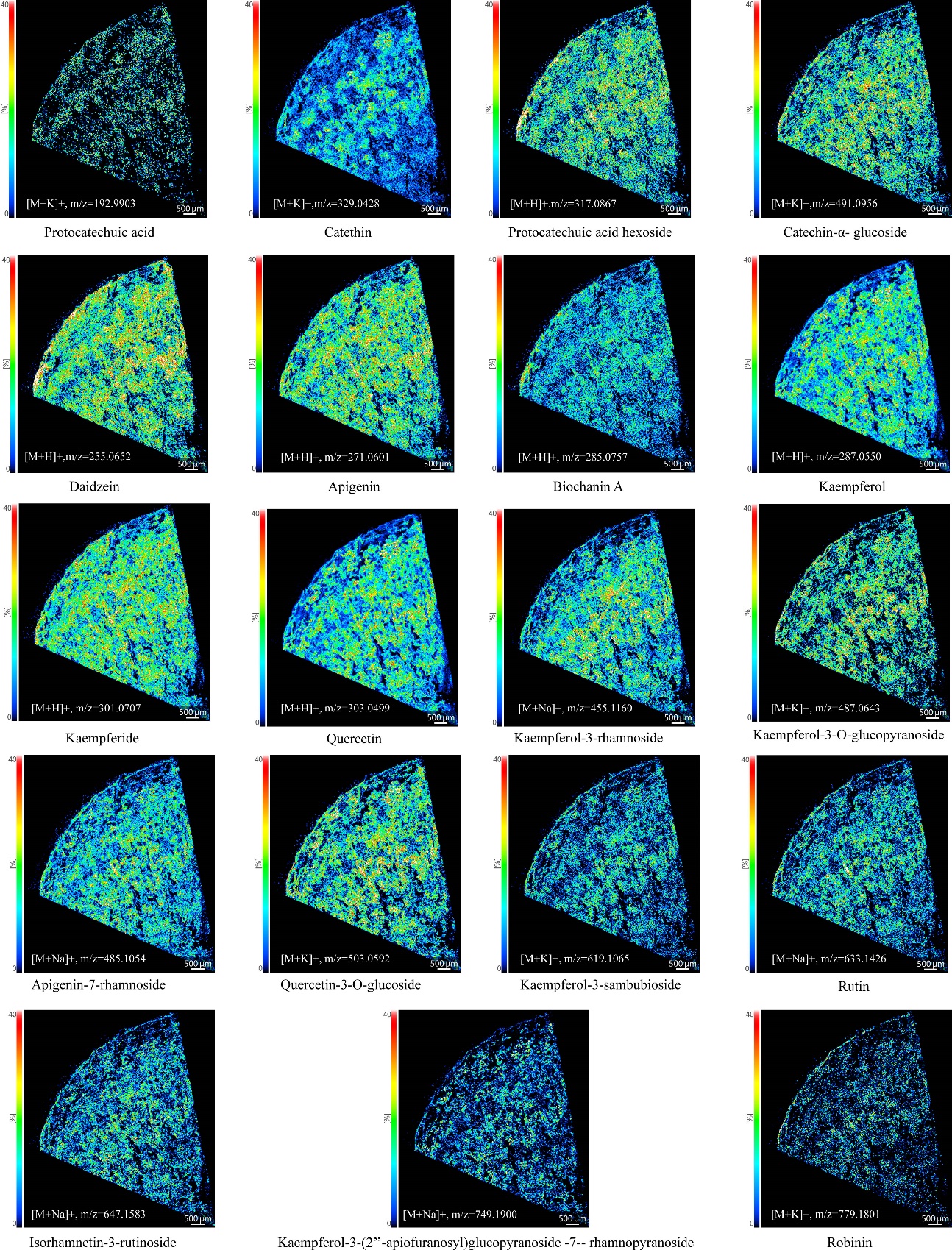


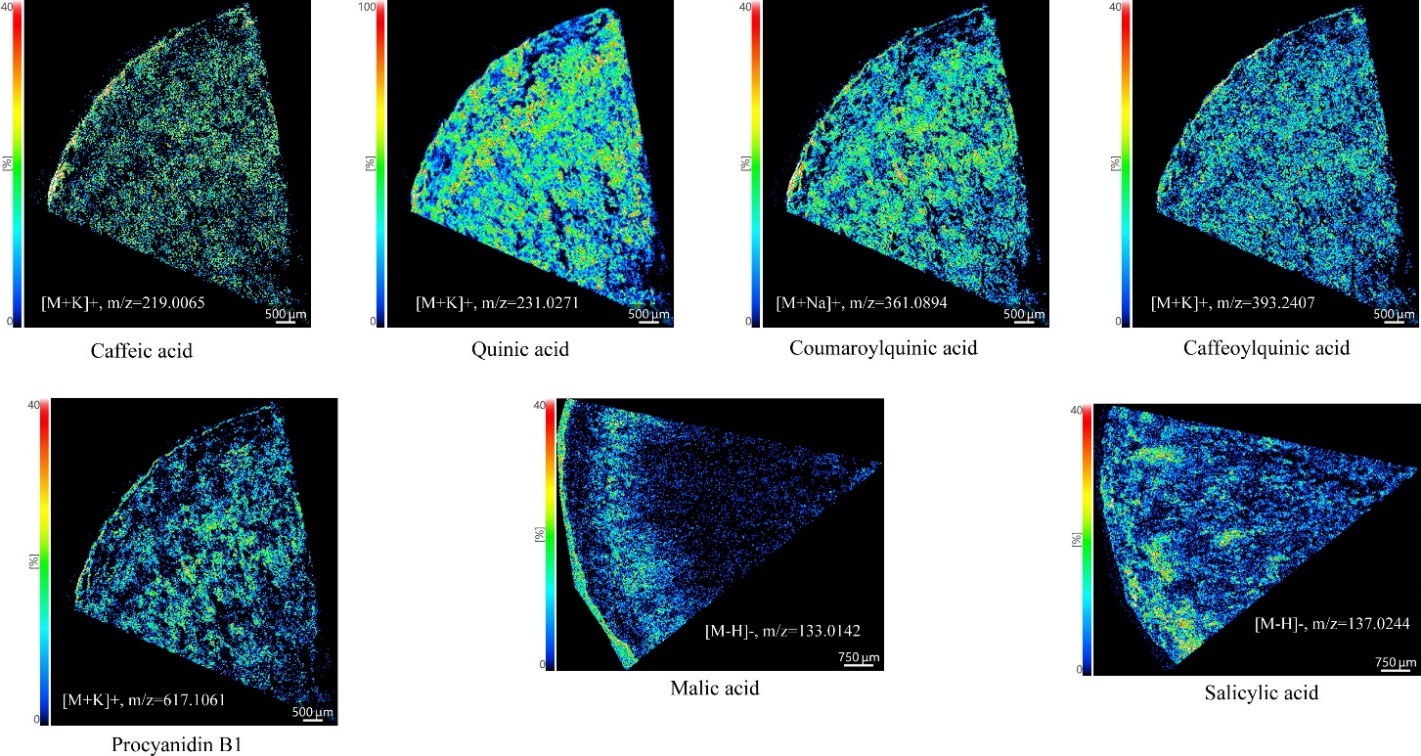


**Supplementary Figure S2.** The MALDI-TOF-MS imaging of 26 compounds in *T. hemsleyanum* on the cross section. (color scale displays ion relative intensity, blue represents low intensity)
